# Women Skin Microbiota Modifications during Pregnancy

**DOI:** 10.3390/microorganisms12040808

**Published:** 2024-04-17

**Authors:** Giulia Radocchia, Francesca Brunetti, Massimiliano Marazzato, Valentina Totino, Bruna Neroni, Giulia Bonfiglio, Antonietta Lucia Conte, Fabrizio Pantanella, Paola Ciolli, Serena Schippa

**Affiliations:** 1 Department of Public Health and Infectious Diseases, Sapienza University of Rome, 00185 Rome, Italy; francesca.brunetti@uniroma1.it (F.B.); m.marazzato79@gmail.com (M.M.); vale.totino@hotmail.it (V.T.); bruna.neroni90@gmail.com (B.N.); giulia.bonfiglio@gmail.com (G.B.); antoniettalucia.conte@uniroma1.it (A.L.C.); fabrizio.pantanella@uniroma1.it (F.P.); serena.schippa@uniroma1.it (S.S.); 2 Policlinico Luigi Di Liegro, 00148 Rome, Italy; 3 Diagnostic Medicine and Radiology, UOC Clinical Pathology, Policlinico Umberto I Hospital, 00161 Rome, Italy; 4 Department of Maternal Infantile and Urological Sciences, Policlinico Umberto I Hospital, Sapienza University of Rome, 00185 Rome, Italy; paola.ciolli@uniroma1.it

**Keywords:** skin microbiota, pregnancy, metabolic pathways

## Abstract

Several studies have shown fluctuations in the maternal microbiota at various body sites (gut, oral cavity, and vagina). The skin microbiota plays an important role in our health, but studies on the changes during pregnancy are limited. Quantitative and qualitative variations in the skin microbiota in pregnant woman could indeed play important roles in modifying the immune and inflammatory responses of the host. These alterations could induce inflammatory disorders affecting the individual’s dermal properties, and could potentially predict infant skin disorder in the unborn. The present study aimed to characterize skin microbiota modifications during pregnancy. For this purpose, skin samples were collected from 52 pregnant women in the first, second, and third trimester of non-complicated pregnancies and from 17 age- and sex-matched healthy controls. The skin microbiota composition was assessed by next generation sequencing (NGS) of the V3–V4 region of the bacterial rRNA 16S. Our results indicate that from the first to the third trimester of pregnancy, changes occur in the composition of the skin microbiota, microbial interactions, and various metabolic pathways. These changes could play a role in creating more advantageous conditions for fetal growth.

## 1. Introduction

In a woman’s life, pregnancy is an exclusive period in which metabolic, hormonal, anatomical, and immunological modifications occur to offer the best milieu for embryonic growth. The outcomes of hormonal changes during pregnancy produce several effects on maternal microbiome, which undergoes fluctuations at different body sites. The most studied are those related to the gut, oral cavity, and vagina [1]. In the first trimester (in a normal pregnancy), gut microbiota looks like that of a non-pregnant woman, characterized by a dominance of *Firmicutes* (particularly *Clostridiales* and *Faecalibacterium prausnitzii*) over *Bacteroides* [2,3,4,5]. Subsequently, gut microbiota composition changes considerably during pregnancy [6], with a continuous decrease in α-diversity and an increase in β-diversity being observed in the second and third trimester [7]. These changes in microbiota diversity were considered a physiological adjustment during pregnancy, appearing to be responsible for the gradual weight gain and insulin resistance [5]. As pregnancy progresses, the gut microbiota becomes progressively enriched with bacteria that stimulates weight gain, energy production and storage, and insulin resistance. These factors are considered beneficial for fetal growth and future lactation [8]. A decrease in butyrate-producing bacteria was also demonstrated in parallel with an increase in Bifidobacteria, Proteobacteria, and lactic acid-producing bacteria [2,6,9]. Moreover, in the third trimester, there is a significant decrease in bacteria (such as *Faecalibacterium prausnitzii*) that produce short-chain fatty acids (SCFAs). The change in butyrate production is linked to low-grade inflammation, decreased insulin sensitivity, and amplified intestinal absorption of essential elements [2,5,8]. The increase in taxa such as *Akkermasia*, *Bifidobacterium,* and *Firmicutes* may also be related to increased energy storage [10]. In addition, the continuous increase of Actinobacteria (mainly Propionibacterium) and Proteobacteria (Enterobacteriaceae, especially *Escherichia coli*) could be helpful in the defense of the maternal–fetal complex against external infections [2,10,11]. Confirming these observations, an in vivo study showed that transplanting gut bacteria derived from women in the third trimester of pregnancy into germ-free mice induced metabolic changes similar to those of gestational diabetes [2]. 

Changes in the vaginal microbiota were also reported during pregnancy [12]. A decrease in the richness and diversity of the vaginal microbiota was reported, likely connected to higher estrogens levels, absence of menstruation, and variation in cervical and vaginal fluid. These factors configure a vaginal habitat with strong and defined selective pressures, which in turn lead to the selection of few microbial taxa (justifying the observed decrease in richness and diversity). In full-term and uncomplicated pregnancies, the composition of vaginal microbiota during gestation remains stable. An increase in microbial diversity is observed before the delivery, and the vaginal microbiota becomes similar to that of non-pregnant women, serving as a trigger for the onset of labor [12]. 

Furthermore, an increase in the quantity of bacteria in the oral microbiota during pregnancy was reported in several studies [13,14,15,16,17], particularly *Porphyromonas Gingivalis*, *Aggregatibacter*, *Actynomycetecomitans*, *Streptococci*, *Staphylococci,* and *Candida* [18,19]. 

Although the gut, vaginal, and oral microbiota appear to be the most relevant in defining health or disease phenotypes in humans, other microbial communities such as the skin microbiota play important roles and influence several human physiological processes [1]. The microbes of the skin microbiota significantly influence human immune function. The skin immune system is composed of a combination of host and microbial characteristics that act in a mutualistic relationship [20]. The skin is the biggest organ in our body and is an important protective barrier from the external environment. Changes in the skin microbiota in pregnant women can play key roles in altering host immune and inflammatory responses. A recent study has shown that alterations in skin microbiota induce inflammatory modifications that may be implicated in changes in skin properties and may predict skin disorders in newborns [21]. Thus, pregnancy can induce both qualitative and quantitative modifications in skin microbial ecosystem. However, studies on changes in the skin microbiota are still limited.

To fill the gap in knowledge of fluctuations in the skin microbiota during pregnancy, the aim of our study was to characterize the skin microbiota during gestation in healthy women who carried an uncomplicated pregnancy to term. For this purpose, our study population consisted of pregnant women in the first, second, and third trimesters, together with a group of non-pregnant, age-matched women as a control group.

## 2. Materials and Methods

### 2.1. Patients Enrolment/Study Design and Participants Population

Patients were enrolled in the Complex Obstetrics Operational Unit of “Policlinico Umberto I” Hospital, Rome, Italy. We recruited a total of 52 Italian pregnant women (median age 33) at different trimesters, and 17 Italian non-pregnant, age-matched women for the control group during the same season (winter). The exclusion criteria were presence of skin and/or systemic disorders and use of topical or systemic antibiotics in the three months preceding sample collection. 

### 2.2. Samples Collection

We decided to study the skin microbiota in pregnancy starting from the instep area to have greater adherence to the study by pregnant women, as this is an easily accessible area. We asked the study participants not to wash or apply treatments for 12 h before sampling. At the time of sampling, skin bacteria were collected from the instep of pregnant and non-pregnant women (Supplementary Figure S1) by the swabbing method with slight modifications [22]. In brief, a 4.4 × 4.4-cm square on the designated area [22] (Supplementary Figure S1) was softly wiped twice with a sterile cotton swab soaked in physiological solution (0.9% sodium chloride) in a Z-stroke manner [23] (Supplementary Figure S1). For both patients and controls, swabs were collected at the same anatomical site, and all collection procedures were performed by a single qualified researcher. After collection, the swabs were stored at −80 °C until processing.

### 2.3. DNA Extraction

DNA extraction was performed with the dedicated Blood and Tissue DNeasy kit (Qiagen, Hilden, Germany), as the manufacturer reported. To collect all bacterial DNA, the swab head was immersed in 200 µL of lysis buffer ATL for 30 min before proceeding to extraction. DNA quality and quantity were checked using the NanoDrop™ 2000/2000c Spectrophotometers (Thermo Fisher Scientific, Waltham, MA, USA). The obtained DNA was normalized to a final concentration of 20 ng/µL for sequencing. The V3–V4 region of the bacterial 16S rRNA gene was amplified and sequenced by next-generation sequencing (NGS) on an Illumina MiSeq 2 × 300 bp platform.

### 2.4. Quality Control of the Sequences and OTU Picking

After demultiplexing, reads were merged using USEARCH v11 [24] with a minimum percentage identity of 85% between aligned sequences. Then, after primer sequences elimination using Cutadapt 2.1 [25], sequences were filtered by Trimmomatic 0.39 [26], setting the following parameters: LEADING:30, SLIDINGWINDOW:5:30, and MINLEN:5. Quality filtered sequences were imported in the software package Quantitative Insights into Microbial Ecology 2 (QIIME2) V22.2 [27] and passed to the Dada2 algorithm [28] for chimera-checking. For all downstream analyses, QIIME2 was employed, except those for which an alternative software package is specified. Operational taxonomic units (OTUs) defined by a 97% of similarity were selected by grouping sequences with an open reference method against the 97% clustered Greengenes rDNA reference database v13_8. To minimize artifacts, OTUs found in one sample and/or having <10 sequences in the entire population were cleaned out.

### 2.5. Alpha and Beta-Diversity Analysis

**Figure 1:**
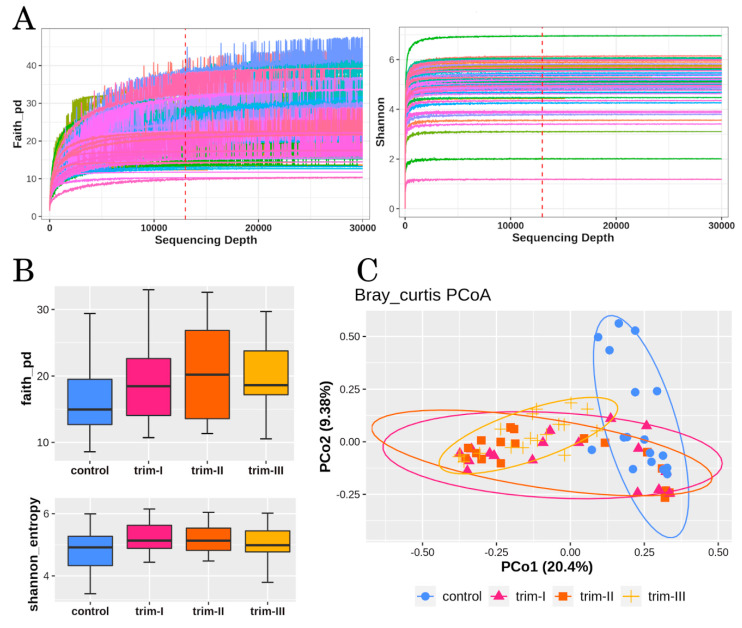
Microbiota diversity analysis. (**A**) Rarefaction curves of 69 samples, based on Faith’s phylogenetic diversity and Shannon diversity indices. Dashed lines indicate rarefaction depth (13,000 reads). (**B**) Box plots showing α-diversity estimators, measured for each group. (**C**) PCoA plot of bacterial β-diversity based on Bray–Curtis dissimilarity according to individual health status. For each group, a 90% confidence interval has been drawn. Numbers between parentheses represent percentage of total variance explained through principal coordinates.

Samples were rarefied to a total of 13,000 reads in agreement to the computed rarefaction curves (Figure 1A). To consider several aspects of diversity, including phylogenetic distance, evenness, and richness, the Shannon index [29] and Faith’s phylogenetic distance [30] were computed for α-diversity, while Bray–Curtis dissimilarity [31] metrics were used for β-diversity. Principal coordinates analysis (PCoA) was performed in the QIIME2 software package for visualization and analysis of the bacterial community within samples.

### 2.6. Taxonomy Assignment

The taxonomy assignment of OTUs was carried out using a Naive Bayes classifier trained on a custom 97% clustered version of the Greengenes rDNA v13_8 reference database, in which the sequences were trimmed to include only the V3–V4 regions. 

### 2.7. Putative Functional Profiling

Functional metagenomic predictions were conducted on the 16S rDNA reads using the Phylogenetic Investigation of Communities by Reconstruction of Unobserved State 2 (PICRUSt2) QIIME2 plugin [32]. The predicted pathways were categorized according to the Metacyc database [33]. 

### 2.8. Network Analysis

For every trimester, a separate correlation network was generated. OTUs having a mean relative abundance of less than 0.01% in the entire population were removed in the first filtering phase. Next, OTUs with a count of zero for each group were removed individually. CoNet v1.1.1 [34], an application that can be found in Cytoscape [35], was used to examine the correlations between the remaining unique entries. In order to overcome the weakness presented by the use of a single metric with regard to compositionality, matching zeros, and sample size, the following combination of methods was used: Pearson correlation, Spearman rank sum correlation, Bray–Curtis dissimilarity, and mutual information. A cutoff threshold of 0.6 was applied for both positive and negative values (−0.6 ≥ correlation ≥ 0.6) for all considered metrics. Retained were relationships that were validated by a minimum of two distinct correlation metrics. Every pair’s statistical significance was examined using 500 row shuffle randomizations and 100 bootstraps. Using Fisher’s approach, the *p*-values pertaining to multi-edges connecting the same pair of nodes were combined, and the merged *p*-values were adjusted for multiple comparisons. To account for compositionality bias, a sample-wise normalizing step was carried out for every item pair in each round of randomization. Each estimated network’s topological properties were determined using Cytoscape’s Network Analyzer plugin. The cluster of nodes with a high degree of positive correlation was identified through the application of the Glay method, which is a feature of the clusterMaker plugin for Cytoscape [36]. Highly linked nodes (HUBs) were found to be present. The presence of HUBs were determined using a degree-based approach, as previously reported [37].

### 2.9. Statistical Analysis

Levene’s test, Mann–Whitney U-test, Kruskal–Wallis test, and Dunn’s post hoc test were performed to determine significant differences with respect to continuous variables. The Analysis of Similarities (ANOSIM) test with 1000 permutations was calculated in QIIME2 on the β-diversity distance matrix to assess the presence of statistically significant partitions between the groups. To test the differences in taxonomic and functional composition between the groups, differential abundance analysis (DAA) was performed using the ANOVA-Like Differential Expression 2 (Aldex2) R package, setting an effect size (ES) cutoff of 1. DAA results will be evaluated, taking in consideration both ES and *p*-value. Data processing, plotting and part of the statistical analyses were performed using R (version 4.0.5) (https://www.r-project.org/) together with the following R packages: “ggplot2” (v3.3.5), “stats” (v4.0.5) and “car” (v3.1.2). The Benjamini–Hochberg false discovery rate (FDR) correction was used to account for multiple hypothesis testing when necessary. In all cases, a *p*-value ≤ 0.05 was considered statistically significant.

## 3. Results

A total of 52 pregnant women were enrolled, of which 17 (32.7%), 17 (32.7%), and 18 (34.6%) were at the first (trim-I), second (trim-II), and third trimester (trim-III) of pregnancy, respectively, while 17 (32.7%) were controls. All 69 samples underwent 16 s rRNA gene-based microbiota analysis. From a total of 7,275,061 sequences passing the filtering bioinformatics processes (median, IQR: 92710, 60,139.25–133,438/samples), 4645 OTUs were identified.

### 3.1. Diversity

No statistically significant differences were found between the groups with respect to the distribution of α-diversity nor its variance (Figure 1B), while statistically significant partitions among groups were found in the β-diversity (Bray–Curtis, *p* = 0.001) (Figure 1C). Pairwise comparisons evidenced differences in the microbiota composition of women at trim-I (R = 0.26, *q* = 0.0020), at trim-II (R = 0.32, *q* = 0.0020), and at trim-III (R = 0.54, *q* = 0.0020), compared to the control group. The ANOSIM statistic “R” compares the mean of ranked dissimilarities between groups to the mean of ranked dissimilarities within groups. Significant differences were also found between trim-I and trim-III (R = 0.08, *q* = 0.0444), as well as between trim-II and trim-III (R = 0.074, *q* = 0.0444). No significant partition was evidenced between trim-I and trim-II.

### 3.2. Taxonomy and Differential Abundance Analysis

**Figure 2:**
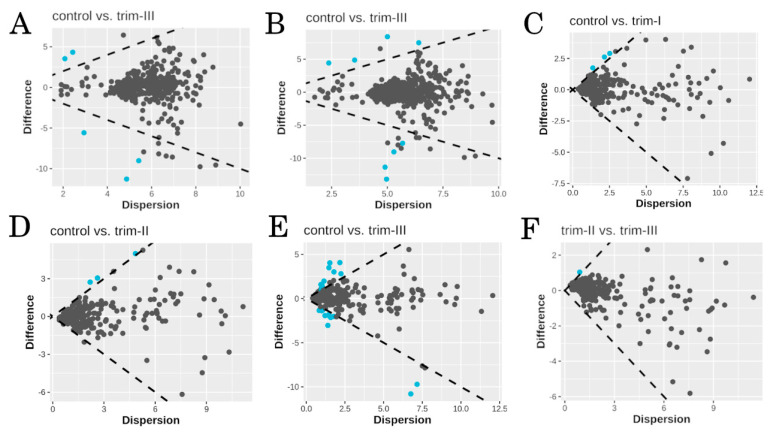
DAA and Aldex2 Effect plot, displaying median difference in pairwise comparisons among groups (difference) vs maximum within-group variance (dispersion). In each plot, taxa or pathways are represented by single dots and differentially abundant taxa or differentially enriched pathways (*p*-value < 0.05 and |ES| > 1) are displayed in light blue. Dashed lines indicate ES threshold. (**A**,**B**) DAA of bacterial taxa at genus and species level, respectively. Negative differences indicate higher abundance in control group. (**C**–**F**) Differential enrichment analysis of Metacyc pathways. Negative differences indicate higher enrichment in control (**C**–**E**) or in trim-II group (**F**).

A total of 578 different genera and 751 bacterial species were determined across all samples. Differential abundant taxa were detected only between women at the third trimester of pregnancy and the controls (Figure 2A,B, Table S1). *Pseudomonas* (*q* ≤ 0.0001, ES = 1.60) and *Janthinobacterium* (*q* ≤ 0.0001, ES = 1.70) were significantly more abundant in woman at trim-III, while a significantly lower abundance of *Sphingomonas* (*q* ≤ 0.0001, ES = −1.94) and *Oscillospira* (*q* = 0.01, ES = −1.54) was found compared to the controls (Figure 2A). At species level, trim-III women were characterized by a significantly higher abundance of *Janthinobacterium lividum* (*q* ≤ 0.0001, ES = 1.36), *Pseudomonas veronii* (*q* ≤ 0.0001, ES = 1.47) and *Pseudomonas fragi* (*q* = 0.038, ES = 1.05), as well as by a significantly lower abundance of *Sphingomonas yabuuchiae* (*q* <0.0001, ES = −2.53) and *Bacteroides plebeius* (*q* = 0.042, ES = −1.20) (Figure 2B).

### 3.3. Functional Profiling 

The pairwise comparisons with the control group revealed the presence of significantly enriched pathways in pregnant women across each trimester, including the superpathway of ornithine degradation (Metacyc ID: ORNDEG-PWY) and polymyxin resistance (Metacyc ID: PWY0-1338), which were the only pathways significantly decreased in all trimesters compared to the controls (Figure 2C–E; Table S2). Notably, the highest number of pathways significantly different from the controls was observed in trim-III. In this context, 11 pathways were found to be enriched in the control group, while 12 were enriched in trim-III (Table S2). Statistically significant results were also found between women in trim-II and trim-III, with methanol oxidation to carbon dioxide (Metacyc ID: PWY-7616) being the only significantly enriched pathway in trim-III (*q* = 0.008, ES = 1.10) (Figure 2F).

### 3.4. Network Analysis

**Figure 3:**
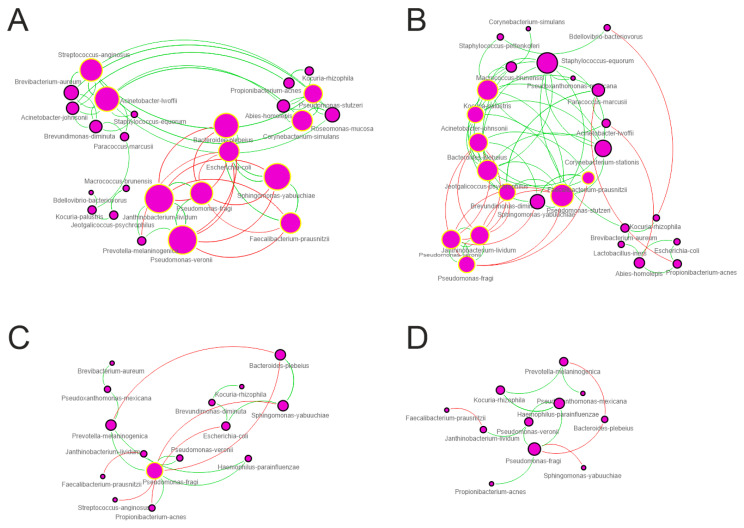
Co-occurrence networks computed at species level. (**A**) Control, (**B**) trim-I, (**C**) trim-II, (**D**) trim-III. Node size is proportional to number of edges departing from node, while edge thickness is proportional to = strength of correlations. Green and red edges represent positive and negative correlation, respectively. Nodes with yellow border are HUBs.

The analysis of microbial interactions represents a powerful approach to study the assembly and function of the microbiota ecosystem. To this aim, we constructed a graphical representation of microbial networks for each group/time-point separately reporting co-occurrence/co-exclusion of species (Figure 3). For each computed co-occurrence network, topological parameters were determined. Obtained results showed that moving from the first to the third trimester microbial networks appear more limited in terms of participants (nodes) and interactions (edges). Particularly for all considered parameter, the microbial network computed for the first trimester presents values very close to those characterizing the control group (Table 1). 

The analysis of clusters present within the computed networks also shows a progressive loss of connectivity as well as changes in the synergism–exclusion ratio. Particularly, the network computed for controls and that determined for the trim-I showed a greater number of highly connected modules while the networks computed for the trim-II and trim-III lost these aggregations of nodes (Figure 3).

Within networks, the highest number of HUB species (no. 11) was observed in the control and the trim-I, although the key species begin to differentiate. A marked reductions of keystone taxa were evidenced at trim-II and trim-III showing 1 and no HUBs respectively (Table S3). Among species, *Sphingomonas yabuuchiae*, *Bacteroides plebeius*, *Janthinobacterium lividum,* and *Pseudomonas fragi* resulted to be HUB species in the control group network, with the last three maintaining this status even at trim-I.

## 4. Discussion

During pregnancy, to accommodate the developing fetus in a woman’s body, important anatomical and physiological changes occur. These changes begin after conception and affect all organs of the body [38]. For uncomplicated pregnancy, these alterations resolve after the gestation period [39]. These changes also involve the pregnant woman’s microbiota. Different studies report that the areas most affected by pregnancy for microbiota composition are the vaginal, intestinal, and oral environments. To date, little is known about the skin microbiota in pregnancy. The skin microbiota has been mainly studied in infants with preterm delivery [40,41,42,43,44]. Our study aimed to assess changes in skin microbiota during the three trimesters of pregnancy, taking the instep as the sampling area. Comparison between the three groups of pregnant women (first, second, and third trimester) and the control group (non-pregnant women) showed no significant differences with respect to the ecological indices evaluated (alpha-diversity estimators). Instead, significantly different partitions in microbiota composition were observed for all three trimesters compared with the control group, as well as between III and I and III and II, but not between I and II trimesters. Changes in the relative abundance of several bacterial taxa were also observed. In particular, the genera *Pseudomonas* and *Janthinobacterium* genera, and the species *Janthinobacterium lividum*, *Pseudomonas veronii,* and *Pseudomonas fragi* were found to be increased in the III trimester compared with the control. While the genera *Sphingomonas* and *Oscillospira*, and species *Sphingomonas yabuuchiae* and *Bacteroides plebeius* were found to be decreased in the III trimester compared to the control group. No differences were found in the relative abundance of taxa in the pregnancy trimesters considered, nor between trimester I and II and the control group. Therefore, our results indicate that changes in the microbial ecosystem of pregnant women also occur at the skin level (as already seen in the intestinal and vaginal districts), making it significantly different from that of non-pregnant women. Within the networks, the highest number of HUB species (No. 11) was observed in the control group and in the first trimester. The species *Corynebacterium simulans*, which is important in the skin ecosystem and particularly found in wetlands and feet [45], was found to be a keystone species in the control group network, although its relative abundance did not differ among the groups considered in our study. Our results indicate that the role played by *Corynebacterium simulans* is lost during pregnancy as early as the first trimester (Figure 3 and Table S2). The number of HUBs, rapidly decreasing during the second trimester (No. 1), tends to be completely absent in the third trimester. Among the species evidenced in the network of trim-III, *Pseudomonas fragi* is the more connected, but not enough to be considered a HUB. Looking at the topological parameters of co-occurrence networks, we can observe the changes that occur during pregnancy in microbial interactions. In fact, along with the marked reduction in keystone species, we can observe a decrease in participants (nodes) and interactions (edges) from the first to third trimester (Table 1). In the second and third trimester networks, the microbes seem to be less connected to each other and the ratio of synergistic to non-synergistic relationships (Syn/Escl ratio) is strongly decreased (Table 1). Thus, a state of dysbiosis is established, as also happens in intestinal and vaginal microbial ecosystems during pregnancy. Although they are more similar in topological parameters, there are also substantial differences between the first trimester and control networks. In particular, important HUB species are lost in the first trimester, indicating changes in the relationships between microbes. Differences were also found in metabolic pathways from in silico analysis. The only pathways found to be significantly depleted in all trimesters compared with controls were: the ornithine degradation superpathway, important for the conversion of L-ornithine to the polyamine putrescine and its subsequent degradation to 4-aminobutanoate, a peculiarity of some bacterial taxa such as *Escherichia coli* that we showed to be a HUB species only in the control network, and the superpathways related to polymyxin resistance (Metacyc ID: PWY0-1338), a class of antibiotics used primarily to treat infections by Gram-negative bacteria, which interfere with the bacterial cell membrane. Ornithine is an amino acid produced as a result of the partial digestion of arginine by the arginase enzyme, leading to the ornithine and urea production.

Ornithine is an amino acid obtained, along with urea, by the partial digestion of arginine by the enzyme arginase. It is a key product of the urea cycle, enabling the elimination of excess nitrogen. Depletion of the ornithine degradation pathway could lead to an increase in this amino acid, which could lead to urea cycle malfunction and nitrogen accumulation. During pregnancy, the body retains nitrogen for the purpose of synthesizing new protein tissues [46]. The other pathway that is decreased during pregnancy is polymyxin resistance. Polymyxins have bactericidal activity against many Gram-negative bacilli and are naturally produced by Gram-positive bacteria to counteract the growth/overgrowth of Gram-negative bacteria in their habitat. Polymyxin B resistance is typically acquired through modifications of the lipid fraction of the outer lipopolysaccharide membrane. These modifications positively charge the cell surface, which loses its affinity for (positively charged) polymyxins. Of course, these modifications can have other effects on the tissue affinity of microbes toward host cells.

In contrast, the pathway of methanol oxidation to carbon dioxide was found to be significantly enriched in trim-III (Figure 2F) compared with trim-II. A recent study reported a correlation among the alteration in the oxidation pathway of methanol to carbon dioxide and the increase in the waist-to-hip ratio (WHR) [47].

## 5. Conclusions 

In addition to the gut microbiota, the skin microbiota also plays a role in maintaining body homeostasis by influencing immune and inflammatory responses. Alterations in the skin microbiota can also induce inflammatory disorders that affect an individual’s dermal properties [21]. Pregnancy is a particular period in a woman’s life when many changes occur. To date, most studies on the skin microbiota during pregnancy were conducted on pre-term pregnancies. In contrast, our study was conducted on women who carried uncomplicated pregnancies to term, with the aim of characterizing the composition of the microbiota during the three trimesters of pregnancy in a normal situation. We demonstrated significant changes in the composition of the skin microbiota of the foot, relationships among microbes, and metabolic pathways during pregnancy. Important species in the skin microbial ecosystem, such as Corynebacterium simulans, seem to lose their importance during pregnancy, while other species usually considered to be related to a dysbiotic microbiota have become more important and thus take on weight in the microbial community during this period. Our results also showed changes in several pathways by in silico approach. Among them, the carbon dioxide pathway was found to be enriched in trim-III compared with trim-II. This pathway was related to increased waist-to-hip ratio by a recent study [47]. This connection could also explain our results, given the increase in waist-to-hip ratio during the pregnancy period. The depletion of the ornithine degradation pathway, which leads to an increase in nitrogen, is related to the actual increase in this element observed in pregnant women, which in turn is linked to the synthesis of new proteins in tissues. Our results reinforce the idea that the observed changes in the skin microbiota during pregnancy contribute to better conditions to promote and protect fetal growth. In addition, they provide more information on how the skin microbiota may influence changes during gestation through its interactions with the host and metabolic pathways, adding important information to better follow pregnancy outcome. However, further investigation is needed to confirm these findings. These initial findings are related to only one district of the skin microbiota, but we are aware that in order to obtain a complete picture of the skin microbiota in pregnancy, other skin districts should be evaluated in further studies.

## Figures and Tables

**Table 1:** Topological parameters of co-occurrence networks computed for each group/time-point.

Topological Parameter	Control	Trim-I	Trim-II	Trim-III
Nodes	25	26	15	11
Edges	83	67	19	13
Edges/node ratio	3.32	2.57	1.27	1.18
Synergisms	60	53	13	9
Exclusions	23	14	6	4
Syn/Escl ratio	2.61	3.78	2.1	2.2
Average number of neighbors	3.76	4.23	2.4	2.1

## Data Availability

All data generated or analyzed during this study are included in this published article (and its Supplementary Files). Raw data are available on NIH Sequence Read Archive (SRA) under the Bioproject ID PRJNA1078078.

## Supplementary Materials

The following supporting information can be downloaded at: https://www.mdpi.com/article/10.3390/microorganisms12040808/s1, Figure S1: Bacterial collection site and Z-stroke manner swabbing method; Table S1: Differential abundance analysis results; Table S2: Functional profiling results; Table S3: Co-occurrence networks information.
