# Supplementary material for: Women Skin Microbiota Modifications during Pregnancy
Source: Microorganisms. 2024 Apr 17;12(4):808. doi: 10.3390/microorganisms12040808 (PMC11051999; doi:10.3390/microorganisms12040808)
Supplement: Supplementary file 1 [file microorganisms-12-00808-s001.zip › Figure S1. Supplementary Data Skin.pdf]

Supplementary Material

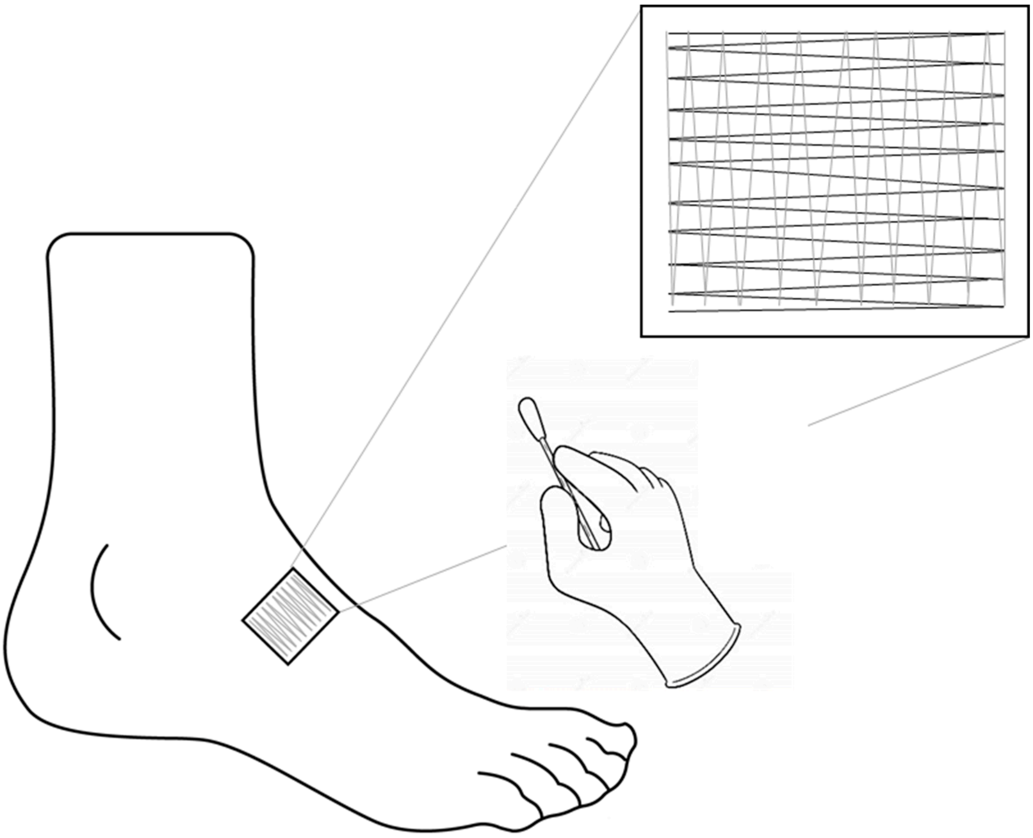

Supplementary Figure S1. Bacterial collection site and Z-stroke manner swabbing method. The sterile wet swab was softly wiped twice, 10x horizontal and 10x vertical, for all sample collected.
